# Clinical Symptoms, Pathogen Spectrum, Risk factors and Antibiogram of Suspected Neonatal Sepsis cases in Tertiary Care Hospital of Southern Part of Nepal: A Descriptive Cross-sectional Study

**DOI:** 10.31729/jnma.5094

**Published:** 2020-12-31

**Authors:** Bijay Raj Pandit, Ashish Vyas

**Affiliations:** 1 Department of Microbiology and Biochemistry, Lovely Professional University, Phagwara, Punjab 144411, India

**Keywords:** *antibiotics*, *bacterial spectrum*, *blood culture*, *neonatal sepsis*

## Abstract

**Introduction:**

Neonatal mortality rate is highest in sub-Saharan Africa and Southern Asia region. The present study is undertaken to find out the prevalence of neonatal sepsis, recognize bacterial pathogens, neonatal risk factors, major symptoms, and their antibiotic sensitivity pattern in neonates in tertiary care hospital in southern Nepal.

**Methods:**

A descriptive cross-sectional study was carried out in a tertiary care hospital from 2^nd^ January 2017 to 20^th^ February 2018 after approval (Ref: 125/2016-17). The sample size was calculated and convenience sampling was done. Data were collected from hospital records and microbiology laboratory and analysed by Statistical Package for Social Sciences.

**Results:**

Out of 1200 clinically suspected cases, early-onset neonatal sepsis was seen in 290 (79.89%). A positive culture was seen in 363 (30.25%) in which maximum bacterial growth was found in 254 (69.98%) males. Preterm gestational age was seen in 265 (73%), low birth weight in 284 (78.23%), a vaginal delivery mode in 279 (76.90%), and delivery in hospital in 232 (63.91%). Likewise *Staphylococcus aureus* in 229 (63.08%) was found maximum followed by *Klebsiella pneumoniae* in 48(13.22%). The major symptom observed was Respiratory distress in 245 (20.41%) while culture positive was seen in poor cry in 94 (53.10%). Mainly effective antibiotics against Gram-positive and gram-negative organisms were Linezolid in 250 (94%) and Imipenem in 46 (90.19%), whereas Penicillin-G in 254 (99.21%) and Ampicillin in 38 (94.74%) found resistance towards organisms respectively.

**Conclusions:**

The high prevalence of neonatal sepsis in our study reflects a huge challenge to reduce the neonatal mortality rate to 12 by 2030 of Sustainable Development Goals. Bacterial isolates exhibited higher resistance towards commonly used antibiotics.

## INTRODUCTION

National Neonatal Mortality rate (NMR) was 21 per 1000 live births and 30 in province two.^1^ Neonatal sepsis is usually divided into early-onset neonatal sepsis(EONS) ≤ 72 hours of life and late-onset neonatal sepsis(LONS) ≥ 72 hours to four weeks of life.^2,3^

The management of neonatal sepsis, the commonest cause, of Neonatal mortality in developing countries is chiefly dependent upon the causative organism, onset of sepsis, site of infection, and related neonatal risk factors.^4^ The immediate treatment with limited information is very challenging to meet the target of Sustainable Development Goals-3(SDG-3) for Nepal.^1^ This requires additional data for developing countries like Nepal due to regional variation.

The study aimed to find out the prevalence of neonatal sepsis in relative to neonatal risk factors along with Antibiotic Sensitivity Pattern (ASP) of the aerobic isolates from neonates in tertiary care hospital in southern Nepal.

## METHODS

This was a descriptive cross-sectionalstudy conducted in the Paediatric ward and NICU from 2^nd^ January 2017 to 20^th^ February 2018 in National Medical College and Teaching Hospital (NMCTH) after approval from the Institutional Review Committee (IRC) of the college (Ref: 125/2016-17). NMCTH is a tertiary care hospital in the southern part of Nepal (province two). Total neonates admitted in the pediatric and neonatal wards were 2965. The present study included all the inborn and outborn neonates with symptoms of sepsis (≤28 days) while excluded those that don't indicate sepsis clinically. All the information regarding age, sex, birth weight, gestational age, mode, and place of delivery, clinical sign, and symptoms were recorded with the help of the pediatric department and hospital records. Among 1200 suspected neonates, blood was collected and processed microbiology laboratory for isolation,identification, and culture sensitivity. The assortment was based on signs and symptoms as described by previous studies.^5,6^ Convenience sampling and the sample size was calculated using the formula.

$$\begin{array}{ll} \text{n} & \text{= }{\text{Z}}^{\text{2}}\times \text{p}\times \text{(1}-\text{p)}/{\text{e}}^{\text{2}}\\ & \text{= }{\left(1.96\right)}^{\text{2}}\times (0.48)\times (0.52)/{(\text{0.03)}}^{\text{2}}\\ & \text{= }1065.40\\ & =1065 \end{array}$$

Where,

n = required sample size
Z = 1.96 at 95% Confidence Interval (CI)
p = prevalence of neonatal sepsis (48%)^7^
e = margin of error (3%).

The total sample size was calculated to be 1065. However, 1200 samples were collected for the study.

1-2 ml of blood was collected with aseptic precaution before administration of starting any antibiotic therapy and carefully transferred into a culture bottle containing Brain Heart Infusion(BHI) Broth immediately to assure the ratio (1:10).^6^

The culture bottles were incubated at 37°C for 7 days aerobically and were observed each day for turbidity, hemolysis of red cells, gas bubbles, and clot formation. During incubation, the first, second, and third subcultures were done on solid media. The axenic isolates obtained from subcultured plates were identified subsequently by standard microbiological techniques like colony morphology, Gram-staining reactions, and biochemical reaction.^8^ The culture bottle did not show growth after 24hr were observed for 7 days before regarded as no growth.^6^ Antimicrobial susceptibility tests were performed of the bacterial isolates to some normally used antibiotics by the Kirby Bauer disk diffusion method and interpreted according to Clinical Laboratory Standards Institute (CLSI) guidelines.^9^ All antibiotic discs used were of Hi-Media, Mumbai, India. Staphylococcus aureus ATCC 25923 and Klebsiella pneumoniae ATCC 700603 were used as control organisms for antibiotic sensitivity testing.

All the data obtained were entered in the Microsoft Excel worksheet and was analyzed using the Statistical Package for the Social Sciences (SPSS) software version (22.0).

## RESULTS

One thousand two hundred blood cultures of neonates were evaluated during the study period. Out of 1200 clinically suspected cases of neonatal sepsis, 1024 (84.33%) were early-onset and 176 (14.67%) were late- onset in which positive blood culture was found in 290 (28.32%) and 73 (41.47%) cases respectively. Neonates ages ranging from 1 to 28 days with a mean age of 2.69±4.39 days. The mode was equal to 1 day and median equal to 1day. Among 1200 enrolled neonates 843(70.25%) were male and 357(29.75%) were female. The male and female ratio of this study was 2.3:1. The occurrence of neonatal sepsis was 363(30.25%). The highest frequency of bacterial growth was seen in 254 (69.98%)male neonates; EONS in 290(79.89%); low birth weight in 284 (78.23%); preterm gestational age in 265 (73%); spontaneous vaginal delivery in 279 (76.86%); delivery in hospital in 232(63.91%) (Table 1).

**Table 1:** Prevalence of positive blood culture in relation to different neonatal risk factors.

Variables	EONS group n (%)	LONS group n (%)	Total n (%)
Neonatal variables			
Gender			
Male	203 (55.92)	51 (14.04)	254 (69.98)
Female	87 (23.96)	22 (6.06)	109 (30.02)
Gestational age at birth			
Preterm(<37weeks)	212 (58.40)	53 (14.60)	265 (73)
Term(>37weeks)	78 (21.48)	20 (5.51)	98 (27)
Birth weight			
<2500gm	232 (63.91)	52 (14.32)	284 (78.23)
≥2500gm	58 (15.98)	21 (5.78)	79 (21.77)
Mode of delivery			
Vaginal	225	54	279 (76.86)
Caesarean section	65 (17.90)	19 (5.23)	84 (23.14)
Place of delivery			
Home	110 (30.30)	21 (5.78)	131 (36.09)
Hospital	180 (49.58)	52 (14.32)	232 (63.91)

**Figure 1:**
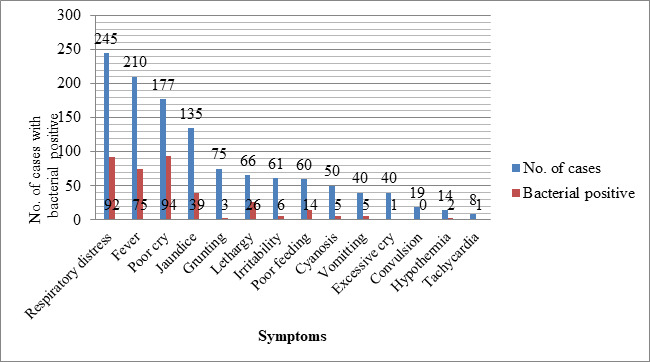
Symptomatic cases with bacterial positive.

The frequent clinical symptoms observed at the time of admission were respiratory distress 245 (20.41%), fever 210 (17.50%), poor cry 177 (14.75%), and the maximum percentage of bacterial growth were seen in poor cry 94 (53.10%) (Figure 1).

Among positive cases, the bacterial spectrum of isolates was shown in Table 2.

**Table 2:** Distribution of isolatec organisms.

Organism isolated	^*^EONS n (%)	^†^LONS n (%)	Total n (%)
Gram-positive organisms	214 (73.80)	56 (76.71)	270 (74.39)
S. aureus	182 (62.76)	47 (64.39)	229 (63.08)
Streptococcus spp.	23(7.93)	5 (6.84)	28 (7.71)
^‡^CONS	9(3.10)	4 (5.48)	13 (3.59)
Gram-negative organisms	76(26.20)	17 (23.29)	93 (25.61)
Klebsiella pneumoniae	41(14.13)	7 (9.58)	48 (13.22)
Pseudomonas aeruginosa	16(5.51)	5 (6.84)	21 (5.79)
E. coli	14(4.82)	3 (4.10)	17 (4.69)
Enterobacter spp.	4(1.39)	2 (2.73)	6 (1.65)
Proteus spp.	1(0.34)	0 (0)	1 (0.27)
Total	290 (79.89)	73 (20.11)	363(100)

* EONS: Early-onset neonatal sepsis

† LONS: Late-onset neonatal sepsis

‡ CONS: Coagulase-negative Staphylococcus

In the present study,the most effective antimicrobial agents against Gram-positive organism was Linezolid 250(94%)Whereas,Penicillin-G 254(99.21%) and Amikacin 190(81.20%) were found resistance. For Gram-negative organisms, Imipenem 46(90.19%), was highly sensitive while antibiotics such as Ampicillin 38(94.74%) and 3rd generation cephalosporin were found resistance towards the isolates in our study. Antibiograms of gram-positive and gram-negative bacteria are shown in (Table 3) (Table 4) respectively.

**Table 3:** Antibiotic sensitivity profile of Gram-positive bacteria.

Antimicrobial	*Staphylococcus aureus* (n=229)	CONS (n=13)	*Streptococcus* spp. (n=1)
Vancomycin (30mcg)	193/226 (85.40)	12/12 (100)	25/28 (89.28)
Linezolid (30mcg)	210/226 (92.92)	12/12 (100)	28/28 (100)
Penicillin-G (10U)	0/225 (0)	0/10 (0)	2/21 (9.52)
Amikacin (30mcg)	29/197 (14.72)	11/12 (91.67)	4/25 (16)
Meropenem (10mcg)	132/169 (78.10)	6/11 (54.54)	3/18 (16.67)
Cefotaxime (30mcg)	10/164 (6.09)	3/9 (33.33)	2/19 (10.52)
Levofloxacin (5mcg)	20/173 (11.56)	4/12 (33.33)	5/19 (26.31)

**Table 4:** Antibiotic sensitivity profile of gram-negative bacteria.

Antimicrobials	*Klebsiella pneumoniae* (n=48)	*Pseudomonas aeruginosa* (n=21)	*E.coli* (n=17)	*Enterobacter* spp. (n=6)	*Proteus* spp. (n=1)
Amikacin(30mcg)	23/47(48.93)	16/19(84.21)	5/8(62.50)	2/5(40)	0/1(0)
Meropenem(10mcg)	19/39(48.71)	16/17(94.11)	5/9(55.55)	4/6(66.67)	1/1(100)
Cefotaxime(30mcg)	0/33(0)	4/13(30.77)	0/5(0)	0/6(0)	0/1(0)
Levofloxacin(5mcg)	33/43(76.74)	14/18(77.78)	12/17(70.59)	5/6(83.33)	1/1(100)
Cefixime(5mcg)	2/33(6.06)	1/7(14.29)	1/5(20)	0/6(0)	0/1(0)
Piperacillin(100mcg)	8/48(16.16)	10/21(47.61)	8/17(47.05)	0/6(0)	0/1(0)
Imipenem(10mcg)	30/32(93.75)	3/4(75)	8/9(88.89)	4/5(80)	1/1(100)
Ceftriazone(30mcg)	4/36(11.11)	3/10(30)	0/5(0)	0/6(0)	0/1(0)
Ceftazidime(30mcg)	4/13(30.77)	4/16(25)	0/17(0)	0/3(0)	0/1(0)
Ampicillin(10mcg)	1/21(4.77)	^*^NT	1/16(6.25)	0/2(0)	0/1(0)

* NT: Not Tested

## DISCUSSION

The causative organisms associated with neonatal sepsis vary from place to place and the episode of the causative organism is dissimilar in different hospitals and even in the same hospital at different times.^7^ The NMR of Nepal is 21/1000 live birth, however, the NMR of Province 2 is thirty.^1^ In this study, the prevalence rate of neonatal sepsis was 30.25% which reflects a high incidence compared to other regions. This finding was approx similar to that of Jain et al. (28.30%), Shrestha et al. (30.85%) from the western and central part of Nepal.^10,11^ respectively and Li et al. (28.26%) from Shanghai, China^12^ but our finding was higher than previously reported by Gyawali et al. (15.1%), Pokhrel et al.(20.7%), Chapagain et al. (14%), Yadav et al. (16.9%), Ansari et al. (12.6%), Shrestha and Subedi et al. (6.1%) from central part Nepal.^5,13–17^ Aku et al. (17.3%) from Ghana, Mehar et al. (22%) from India.^6,18^ On the other hand, our finding was lower than the report of Thapa et al. (37.12%), Lakhey et al. (48%), from Nepal.^7,19^ Kayange et al. (39%) from Tanzania, Zakariya et al. (41.6%) from India, Ullah et al. (57.1%) Pakistan, Gatabelew et al. (77.9%) Ethiopia.^4,20–22^ Inconsistent in a positive rate of blood culture in different studies might be due to different reasons like the variation in culture methods, study plan, administration of prior antibiotics from the primary center, unsuccessful control of hospital-acquired infection.^5,13^ Male neonates (70.25%) enrolled more frequently than females. The same type of neonatal admission was also found in previous studies, Aku et al. (60.7%), Gatabelew et al. (58.2%), Lakhey et al. (56 %), Shrestha and Subedi et al. (63.7%), Ansari et al. (61.4%).^6,4,7,17,16^ This is probably due to the prevalent custom of taking more care of male babies for health concerns in our society.^7^

The study has shown a male-female ratio of 2.3:1 similar to the other studies.^10,23,17,16^ but the result was lower in another study.^11,7^ Thus male babies were suspected to double of neonatal sepsis than female babies due to X-linked immune regulatory gene factor resulting in the host's vulnerability to infections in males.^24^ From the clinically suspected cases of EONS and LONS blood culture positive was found higher in LONS (41.47%) than EONS which is in harmony with the study of Kayange et al. (51.4%)^20^ but different with other studies.^6,10^

The majority of culture-positive sepsis was found in our study within Male neonates(69.98%) which is comparativelyhigher thanother various studies ranging from(52.3%-63.5%)^4,12–15,18,19,22,25^ but Kayange et al. reported the opposite.^20^

In the present study, the incidence of EONS(79.89%) was higher which is in agreement with previous finding (57.1%-91.39%)^10,13,18,19,22,25^ whereas, in the study of Chapagain et al. (83.3%), Yadav et al. (71.2%), Li et al.(52.79%), Kayange et al.(45.31%) prevalence of LONS was higher.^12,14,15,20^ Since causative agents in neonatal EONS acquired from mother during birth play role in early infection.^16^

We find out preterm(73%) neonates are more susceptible to sepsis which is consistent with various authors' findings^13,15,18,19,25^ in contrast to this finding term baby are more prone to sepsis.^4,7,12,14^ Birth of baby before 37 weeks of gestational age might have a poor capacity to increase neutrophil production in accordance to demand to overcome the problem associated with neonatal bacterial sepsis.^26^

Probably due to the unhygienic condition of the vagina during birth and carelessness about the safety precaution of medical personnel who assist the delivery, the baby who delivered by normal vaginal route has a higher rate of sepsis (76.90%) similar to the other findings of different researchers.^4,13,18,19,20,25^ dissimilarity was observed in the study of Yadav et al. and Li et al.^15,12^ This study found out that the baby who delivered in a hospital setting privileged from sepsis(63.91%) as in the finding of Gatabelow et al.(75.79%) and Kayange etal.(69.80%).^4,20^ Here in our study it may be due to the lack of proper environmental monitoring of the hospital and delivery room.

Low birth weight neonates(78.23%) were found more prone to sepsis as compared to normal birth weight babies these findings are tandem with the study of different authors.^7,13,15,19,22,25^ whereas, authors found the opposite.^4,12,14,20^ Maturation of Immunological barriers starts around 32-34 weeks of gestational age and accelerated after birth. So that the level of mucosal antibody is lower in underweight neonates to combat with bacterial antigens.^27^

The major clinical manifestations of our study were respiratory distress(20.41%), fever(17.50%), poor cry(14.75%) which is the findings of others study.^10,12,13,19^ In our study maximum percentage of bacterial growth sepsis was seen in poor cry followed by lethargy, respiratory distress, and fever these findings were similar to other studies.^12,19^

The bacterial profile exposed that the higher incidence of *S. aureus,* followed by *K pneumoniae, Streptococcus spp, Pseudomonas aeruginosa, E. coli, CONS, Enterobacter spp.,* and *Proteus spp.* These bacterial isolates are the predominant causative agent for neonatal sepsis identified by several studies.^4–7,10–23^

The result indicates that gram-positive bacteria 270/363 (74.39%) has preponderance over the gram-negative bacteria 93/363(25.61%) the similar findings were also obtained by Ansari et al.(63.8%), Khanal et al.(73%) Lakhey et al.(72.3%), from Nepal.^7,16,28^ Aku et al.(69%) from Ghana, Geyesus et al.(67.5%) from Ethiopia.^6,25^ It indicates that the majority of the infections were transmitted from medical personnel, relatives because S. aureus is ubiquitous in nature and the major normal flora of skin and nose, careless about the hygiene who care neonates and manipulation of peripheral intravenous lines set up on neonates may acquire these bacteria.^6^ However, a study from Pokhrel et al.(77%), Gyawali et al.(55.9%), Kayange et al.(61.1%), Mehar et al.(56.7%),Yadav et al.(54%), Ullah et al.(78.6%), Zakariya et al.(82%) found gram-negative bacilli predominant over gram-positive cocci.^13,5,20,18,15,22,21^

We found S. aureus and K. pneumoniae was the most common isolate in neonatal sepsis accounting for 63.08% and 25.61% among total isolates. This finding was similar to the finding of other studies.^5,28,18,11,23,25^ and unlike in these studies.^16,13,10,21^ The etiological agent varies due to the environmental condition of the hospital, sanitation of medical personnel, and geographical area.

The single predominant *S. aureus* was found in both onset of sepsis as similar to the other study.^28,25^ but in contrast to this, a different organism was found in the different onset of sepsis.^6,16,12^ In our study the most effective antibiotics against major isolate S. aureus, CONS, and Streptococcus spp. were Linezolid and vancomycin similar to the previous study.^18,12,13^ As observed from the current study, For Klebsiella pneumoniae and other Gram-negative bacteria meropenem, imipenem and Levofloxacin be the drug of choice for treatment of neonatal sepsis. Gram-negative isolates showed a high degree of resistance to commonly used antibiotics such as ampicillin, Piperacillin as well as 3^rd^ generation Cephalosporin. This study agreed with studies.^11,16,5,28^ from Nepal. The liberal use of broad-spectrum antibacterial increases the risk of acquisition of pathogens by interfering with the development of normal flora.^29^

## CONCLUSIONS

Overall, Neonatal sepsis is a life intimidating condition. So, the knowledge of major symptoms, care about associated risk factors, prevalent etiological organism, and current effective antibiotics should be known. Respiratory distress was the major symptom and common risk factors were vaginal delivery in the hospital, birth weight less than 2.5 kg, and a gestational period of fewer than 37 weeks. We found S. aureus was the most common isolate in both onsets of sepsis. We strongly recommend medical personnel working in NICU should be trained and maintain hand hygiene with proper disinfection during the procedure and use of Linezolid and Imipenem against gram-positive and gram-negative bacteria respectively as compared to the broad-spectrum antibiotics which are more rampantly used nowadays. This vital information helps Neonatologist to manage and treat neonates with sepsis. Thus, reduction in mortality will reduce the NMR in the southern part along with overall in Nepal to meet the SDG-3.
